# Ischemic Postconditioning Alleviates Neuronal Injury Caused by Relief of Carotid Stenosis in a Rat Model of Cerebral Hypoperfusion

**DOI:** 10.3390/ijms131013338

**Published:** 2012-10-18

**Authors:** Chunsheng Feng, Tianfei Luo, Li Qi, Boyu Wang, Yinan Luo, Pengfei Ge

**Affiliations:** 1 Department of Anesthesiology, First Bethune Hospital of Jilin University, Changchun 130021, China; E-Mail: fcs1971@hotmail.com; 2 Department of Neurology, First Bethune Hospital of Jilin University, Changchun 130021, China; E-Mail: luotianfei2003@yahoo.com.cn; 3 Department of Neurology, Affiliated Hospital of Guilin Medical College, Guilin 541001, China; E-Mail: drqili009@gmail.com; 4 Department of Neurosurgery, First Bethune Hospital of Jilin University, Changchun 130021, China; E-Mails: drboyuwang@yahoo.cn (B.W.); Yinanluo@gmail.com (Y.L.)

**Keywords:** carotid stenosis, neuronal injury, ischemic postconditioning, oxidative stress, inflammatory response

## Abstract

The effects of early relief of heavy bilateral carotid stenosis and ischemic postconditioning on hippocampus CA1 neurons are still unclear. In this study, we used a rat model to imitate severe bilateral carotid stenosis in humans. The rats were divided into sham group, carotid stenosis group, stenosis relief group and ischemic postconditioning group. Ischemic postconditioning consisted of three cycles of 30 s ischemia and 30 s reperfusion. The cerebral blood flow was measured with a laser Doppler flowmeter. Neuronal death in the CA1 region was observed by hematoxylin-eosin staining, and the number of live neurons was assessed by cell counting under a light microscope. The levels of oxidative products MDA and 8-*iso*-PGF2α, inflammatory factors IL-1β and TNF-α, and the activities of anti-oxidative enzymes SOD and CAT were assayed by specific enzyme-linked immunosorbent assay (ELISA) kits, respectively. We found that relief of carotid stenosis and ischemic postconditioning could increase cerebral blood flow. When stenosis was relieved, the percentage of live neurons was 66.6% ± 6.2% on day 3 and 62.3% ± 9.8% on day 27, which was significantly higher than 55.5% ± 4.8% in stenosis group. Ischemic postconditioning markedly improved the live neurons to 92.5% ± 6.7% on day 3 and 88.6% ± 9.1% on day 27. Further study showed that, neuronal death caused by relief of stenosis is associated with increased oxidative stress and enhanced inflammatory response, and the protection of ischemic postconditioning is related to inhibition of oxidative stress and suppression of inflammatory response.

## 1. Introduction

Carotid artery stenosis remains a major public health issue in the developed countries [1]. In particular, severe carotid stenosis (>70%) often leads to symptoms related to transient ischemic attack and ischemic stroke [2]. Clinical studies have also shown that patients with carotid stenosis would have cognition dysfunction due to long-term cerebral hypoperfusion [3,4]. Animal study demonstrated that almost 50% of the neurons in rat hippocampus CA1 region would die at 30 days when severe bilateral carotid artery stenosis was produced [5]. By contrast, clinical observations and animal experiments demonstrated that the damaged neurological function would be improved when carotid stenosis was relieved [6,7].

Currently, revascularization of the stenotic artery has become the first choice of treatment for the patients with severe carotid stenosis [8]. The time window recommended for carotid endarterectomy is 14 days for patients suffering minor stroke or TIA due to severe carotid stenosis [9], which is also refered for carotid stent palcement. However, recent clinical report showed that early revascularization within 7 days can be safely performed and is preferred over delaying operative treatment in the symptomatic carotid stenosis patients without evidence of intracerebral hemorrhage, carotid occlusion, or permanent neurologic deficits [10]. Moreover, it was thought that cognitive impairment in chronic cerebral hypoperfusion rat models was due to the cerebral hypoperfusion at early stage [11]. Thus, we speculate that early relief of carotid stenosis would benefit hippocampus CA1 neurons which are responsible for cognitive function. Therefore, in this study, we used rat to establish the model of severe carotid stenosis and investigated the effects of early relief of carotid stenosis on CA1 neurons that are vulnerable to reperfusion.

Ischemic postconditioning is emerging as an effective method to protect organ injury. It is defined as a series of rapid intermittent interruptions of blood flow in the early phase of reperfusion that mechanically alters the hydrodynamics of blood flow [12]. Animal studies showed that ischemic postconditioning could prevent neuronal death caused by either global or focal ischemia and reperfusion [13,14]. Additionally, it was found that ischemic postconditioning have protective effects on ischemic injury in heart, kidney, liver and intestine [15–18]. Studies from humans also demonstrated that it could protect human tissues or organs from injury [19,20]. In addition, ischemic postconditioning could effectively regulate the cerebral blood flow to the ischemic region. Wang *et al*. reported that ischemic postconditioning blocked the hyper-perfusion or hypo-perfusion following ischemia/reperfusion, indicating ischemic postconditioning could recover cerebral auto-regulation to blood flow [14]. Ischemic postconditioning could be performed before re-establishment of blood supply to brain, which makes it become a feasible method that could be used potentially in future clinical practice. More importantly, no reports have proved that ischemic postconditioning had any negative effects thus far. The underlying mechanism of ischemic postconditioning has been investigated and was found to be associated with inducing endogenous protective effects, including suppression of oxidative stress, activation of signal pathway, modulation of enzyme activity and inhibition of endoplasmic stress [21–25]. Nevertheless, whether or not ischemic postconditioning has protective effects during the course of relieving carotid stenosis is still unclear, which will also be investigated in this study.

## 2. Results and Discussion

### 2.1. Ischemic Postconditioning Increased Cerebral Blood Flow

**Figure 1:**
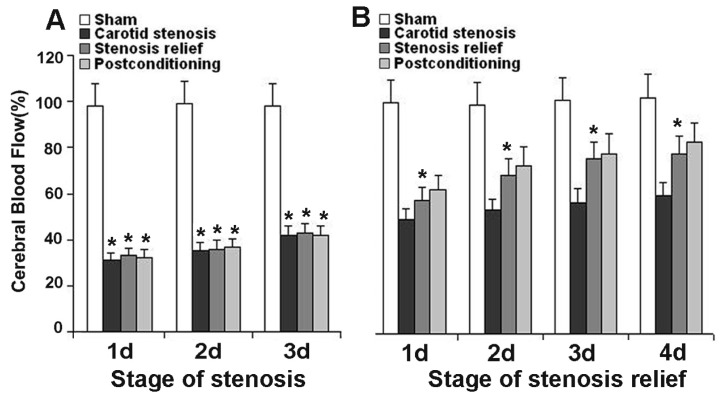
Measurement of cerebral blood flow. (**A**) stage of carotid stenosis; (**B**) stage of stenosis relief. At carotid stenosis stage, cerebral blood flow (CBF) reduced markedly in each group when compared with that in sham group. However, when carotid stenosis was relieved, the CBF increased significantly in comparison with that in the stenosis group at indicated time point. Moreover, ischemic postconditioning improved CBF further, but there is no significant difference between stenosis relief group and ischemic postconditioning group at each corresponding time point. (A: * *p* < 0.01, *vs.* sham group; B: * *p* < 0.01, *vs.* carotid group)

As shown in Figure 1, the cerebral blood flow (CBF) was stable in the sham group, and there was no significant difference at any scheduled time point. When carotid stenosis was maintained, despite the values of CBF could increase from day 1 to day 3, they were still significantly lower than that of the sham group (Figure 1A). By contrast, when the stenosis was relieved, the values of CBF increased markedly in comparison with those in the stenosis group from day 1 to day 4 (Figure 1B). Despite ischemic postconditioning improved the values of CBF, there is no significant difference in CBF between the two groups treated with and without ischemic postconditioning. These results indicated that relief of carotid stenosis could increase CBF, but no significant changes could be found in CBF when ischemic postconditioning is administrated.

### 2.2. Ischemic Postconditioning Rescued Neuronal Death Caused by Stenosis Relief

**Figure 2:**
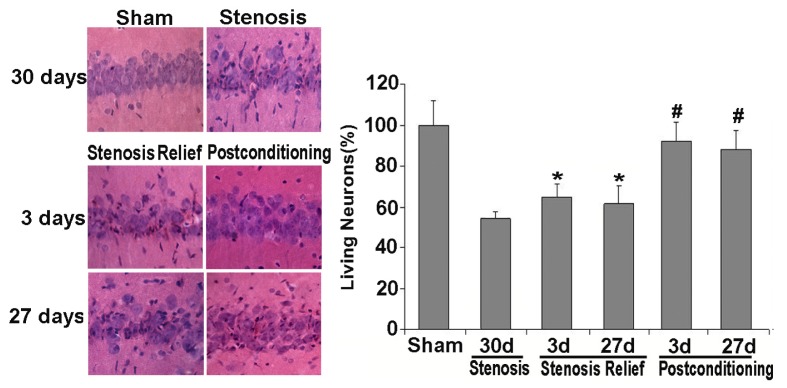
Histological examination of neuron death via hematoxylin-eosin (HE) staining and quantitative analysis of live neurons. When bilateral carotid stenosis was maintained, 55.5% ± 4.8% of the CA1 neurons was found to be alive at day 30. Despite the dead neurons appearing on day 3, the percentage of live neurons was 66.6% ± 6.2% on day 3 and 62.3% ± 9.8% on day 27 when carotid stenosis was relieved. By contrast, when ischemic postconditioning was administrated prior to carotid stenosis relief, the percentage of the live neurons was significantly improved to 92.5% ± 6.7% on day 3 and 88.6% ± 9.1% on day 27, respectively. * *p* < 0.01 *vs.* stenosis group; ^#^ *p* < 0.01 *vs.* stenosis relief group.

HE staining and histological examination is an effective and easy way to examine cellular viability. Generally, compared with the normal neurons, the dead or dying neurons present polygonal condensed nucleus and pink cytosl [26]. In this study, as shown in Figure 2, we found neuronal death or injury appeared on day 30 when bilateral carotid stenosis was maintained, and only 55.5% ± 4.8% of the CA1 neurons were alive. When carotid stenosis was relieved, despite the dead neurons appeared on day 3, the percentage of live neurons was 66.6% ± 6.2% on day 3 and 62.3% ± 9.8% on day 27. Moreover, there was no significant difference in the percentage of live neurons at these two time points. By contrast, when ischemic postconditioning was exerted prior to carotid stenosis relief, the percentage of live neurons was markedly improved to 92.5% ± 6.7% on day 3 (*p* < 0.01 *vs.* stenosis relief group) and 88.6% ± 9.1% on day 27 (*p* < 0.01 *vs.* stenosis relief group), respectively. Thus, ischemic postconditioning consisting of three cycles of 30 s ischemia and 30 s reperfusion was demonstrated to be a protective procedure. Therefore, in the subsequent study, we focused on the prior three days following the relief of carotid stenosis to investigate the mechanism that was responsible for neuronal death and the protective effects of ischemic postconditioning.

### 2.3. Ischemic Postconditioning Inhibited Oxidative Stress Caused by Stenosis Relief

**Figure 3:**
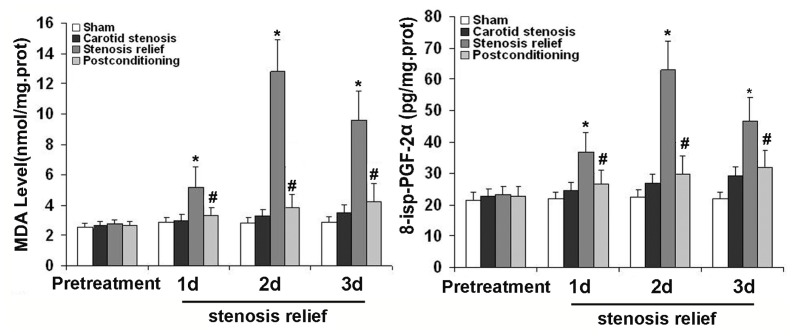
Measurement of the level of oxidized products. At day 1, day 2 and day 3 following the relief of carotid stenosis, the levels of malondialdehyde (MDA) were 5.22 ± 1.31, 12.83 ± 2.11 and 9.62 ± 1.93 times, and 8-*iso*-PGF2α were 36.79 ± 6.18, 62.93 ± 9.42 and 46.58 ± 7.36 times higher than those in sham and carotid stenosis groups, respectively. By contrast, after being treated with ischemic postconditioning, the levels of MDA decreased to 4.12 ± 0.51, 5.49 ± 1.47 and 5.45 ± 1.26 times, and 8-*iso*-PGF2α decrease to 30.13 ± 4.32, 35.85 ± 6.72 and 33.87 ± 4.47 times, respectively. * *p* < 0.01 *vs.* sham group and stenosis group; ^#^ *p* < 0.01 *vs.* stenosis relief group.

To explore whether the neuronal death caused by relief of stenosis and the protection of ischemic postconditioning is associated with oxidative stress, we compared the levels of malondialdehyde (MDA) and 8-*iso*-prostaglandin F2α (8-iso-PGF2α) in the CA1 neurons. As shown in Figure 3, when carotid stenosis was relieved, the levels of MDA and 8-*iso*-PGF2α were significantly elevated from day 1 to day 3 in comparison with those in sham group and carotid stenosis group. However, ischemic postconditioning suppressed their elevation at each scheduled time point. The results suggested ischemic postconditioning could rescue neuronal damage caused by relief of carotid stenosis via inhibition of oxidative stress.

**Figure 4:**
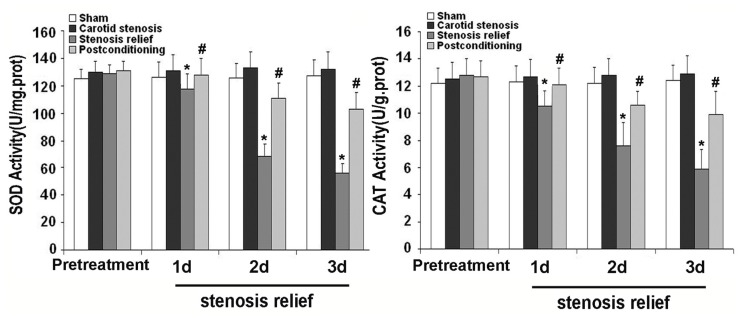
Measurement of the activities of anti-oxidative enzymes. In comparison with those in sham and carotid stenosis groups, the activities of superoxide dismutase (SOD) decreased to 120.98 ± 11.53, 68.26 ± 8.75 and 56.33 ± 6.72 U/mg·protein, and catalase (CAT) reduced to 11.96 ± 1.08, 7.64 ± 1.69 and 5.9 ± 1.45 U/mg·protein at day 1, day 2 and day 3, when carotid stenosis was relieved. However, ischemic postconditioning maintained the activities of SOD to 127.42 ± 12.18, 111 ± 10.76 and 103.66 ± 12.07 U/mg·protein, and CAT to 12.1 ± 1.17, 10.6 ± 1.03 and 9.95 ± 1.68 U/mg·protein. * *p* < 0.01 *vs.* sham group and stenosis group; ^#^ *p* < 0.01 *vs.* stenosis relief group.

Intracellular ROS is mainly cleared by anti-oxidative enzymes such as SOD and CAT, thus we assayed the activities of SOD and CAT in the CA1 neurons pretreated with or without ischemic postconditioning prior to relief of carotid stenosis. As Figure 4 showed, when compared with those in sham and carotid stenosis groups, the activities of SOD and CAT decreased markedly from day 1 to day 3 following the relief of carotid stenosis. However, their reduction at each corresponding time point was inhibited by ischemic postconditioning. This indicated that ischemic postconditioning could significantly improve the activities of SOD and CAT that were damaged by the relief of carotid stenosis.

### 2.4. Ischemic Postconditioning Alleviated Inflammatory Response Caused by Stenosis Relief

**Figure 5:**
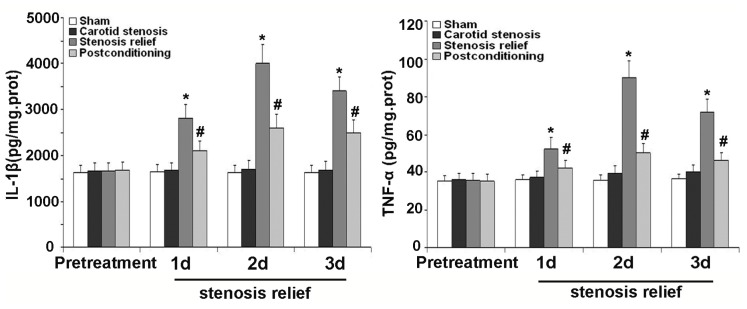
Measurement of the level of inflammatory factor. Compared with the values of sham operated group and carotid stenosis group, protein levels of IL-1β and TNF-α increased respectively to 2800 ± 310, 4000 ± 420 and 3400 ± 310 pg/mg·protein, and 52.66 ± 6.12, 90.18 ± 8.87 and 72.36 ± 6.79 pg/mg·protein at days 1, 2 and 3 after bilateral carotid artery stenosis was relieved. Nevertheless, treatment with ischemic postconditioning decreased the expression of IL-1β and TNF-α to 2100 ± 220, 2600 ± 290 and 2500 ± 270 pg/mg·protein, and 42.09 ± 3.87, 50.15 ± 4.77 and 46.65 ± 4.06 pg/mg·protein. * *p* < 0.01 *vs.* sham group and stenosis group; ^#^ *p* < 0.01 *vs.* stenosis relief group.

To make sure the influence of inflammatory response on neuronal death and protective effects of ischemic postconditioning, we examined the concentration changes of inflammatory factor IL-1β and TNF-α in the hippocampus CA1 neurons treated with or without ischemic postconditioning before the relief of carotid stenosis. As shown in Figure 5, protein levels of IL-1β and TNF-α increased significantly at day 1, 2 and 3 after bilateral carotid artery stenosis was relieved, when compared with the values of sham group and carotid stenosis group. Nevertheless, their expression decreased significantly when the rats were treated with ischemic postconditioning. This indicated that inflammatory response involves in the pathological course of neuronal death caused by relief of carotid stenosis and it could be alleviated by ischemic postconditioning.

### 2.5. Discussion

The model of bilateral carotid artery stenosis used in this study has been proposed for a long time [7,27]. Binding stainless microtube with different diameter to bilateral carotid artery could produce mild, moderate and heavy carotid stenosis in rats [5]. Angiography demonstrated that a microtube with a diameter of 0.45 mm could lead to heavy carotid stenosis (the inner diameter of carotid artery <70%) when it was bound to carotid artery [5]. Thus, as described previously, we used such microtubes in this study to produce heavy carotid stenosis in rats to imitate similar conditions in humans, and examined the destiny of hippocampus CA1 neurons treated with or without ischemic postconditioning before carotid stenosis was relieved. We found that early relief of carotid stenosis could increase live neurons in the CA1 region. Moreover, ischemic postconditioning improved further live neurons and this protection was associated with inhibition of oxidative stress and alleviation of inflammatory response.

Given that hypoperfusion due to long periods of heavy carotid stenosis could result in neuronal injury in CA region at day 30 [5], we measured the changes of cerebral blood flow prior to and after relief of carotid stenosis. We found that the cerebral blood flow recovered from 45% at 3 days of carotid stenosis to 78% at day 4 when heavy carotid stenosis was relieved, which was consistent with previous findings [7]. Because a previous study showed that CBF did not change significantly thereafter [7], we did not measure further the changes of cerebral blood flow. We also found that, when bilateral carotid stenoses were relieved, the percentage of the live neurons was 66.6% ± 6.2% at day 3 and it did not decrease significantly at day 27. By contrast, only 55.5% ± 4.8% of neurons in the CA1 region was found to be alive at day 30 when bilateral carotid stenosis were maintained, despite no neuronal death was found at 3, 7, 14 days (data not shown), respectively. Considering that hypoperfusion had protection on acute neuronal injury when blood supply to brain was re-established [28], we speculate that the neuronal death following relief of carotid stenosis might be due to the sudden increment of cerebral blood flow, which is similar to the condition of acute ischemia and reperfusion. Therefore, our results indicate that early relief of carotid stenosis contributes to increment of cerebral blood flow and protection of neuronal injury in CA1 region, despite it would lead to acute neuronal death in small quantities.

The protective effects of ischemic postconditioning on ischemic injury have been documented in many organs. It was found that both rapid and delayed ischemic postconditioning could exert protection on neuronal injury or death [29,30]. Meanwhile, it was reported that limb remote ischemic postconditioning could protect against focal ischemia as well [31]. Moreover, researchers tested the effects of various modes of ischemic postconditioning on neuronal injury caused by ischemia and reperfusion [14], and found that three cycles of 30 s ischemia and 30 s reperfusion had the most effective protection for neuronal death in CA1 region caused by global ischemia and reperfusion [14,22]. In this study, we found that three cycles of 30 s ischemia and 30 s reperfusion administrated prior to re-establishment of cerebral blood supply significantly protected neuronal injury due to relief of carotid stenosis, and made the percentage of live neurons improve to 92.5% ± 6.7% on day 3 (*p* < 0.01 *vs*. stenosis relief group) and 88.6% ± 9.1% on day 27 (*p* < 0.01 *vs*. stenosis relief group), respectively. Thus, our data indicate that, in combination with ischemic postconditioning, early relief of carotid stenosis would benefit the injury of CA1 neurons. Despite it was reported that ischemic postconditioning could block the hyper-perfusion or hypo-perfusion following ischemia and reperfusion [14], our data showed that there was no significant difference in the CBF at any corresponding time point between the groups pre-treated with and without ischemic postconditioning. Therefore, our results suggest that the protection of ischemic postconditioning is not correlated with the increment of cerebral blood flow.

Since relief of heavy carotid stenosis led to neuronal death in CA1 region at day 3, we focused on the prior three days to investigate the mechanism underlying neuronal death caused by early relief of carotid stenosis and the protective effects of ischemic postconditioning. Animal experimental revealed that oxidative stress is one of the crucial factors leading to neuronal injury or death [22]. Moreover, clinical study also demonstrated that oxidative stress in ischemic stroke played an important role in the pathogenesis of brain injury [32–34]. Oxidative stress is caused mainly by the imbalance between the production of reactive oxygen species (ROS) and reduction of anti-oxidant defense systems [35]. Moreover, brain is an organ prone to produce reactive oxidative species such as superoxide anion (O_2_^−^), hydrogen peroxide (H_2_O_2_), and the hydroxyl radical (OH^−^), because it needs a large amount of oxygen to maintain its normal function but is deficient in anti-oxidants [36].

In oxidative stress, reactive oxygen species (ROS) could damage macro-molecules such as lipid and nuclear acid to generate oxidized products MDA and 8-*iso*-PGF2α [37]. *In vivo*, superoxide dismutase and catalase collaborated to modulate the quantity of reactive oxidative species. Superoxide dismutase converts superoxide anion into hydrogen peroxide, which is then transformed into water and oxygen through catalase [37]. Therefore, in our study, we compared the level of MDA and 8-*iso*-PGF2α, and the activities of superoxide dismutase (SOD) and catalase (CAT) under the condition with or without ischemic postconditioning when carotid stenosis was relieved. Our results showed that MDA and 8-*iso*-PGF2α increased significantly, but the activities of SOD and CAT reduced at any indicated time point when carotid stenosis was relieved. By contrast, ischemic postconditioning inhibited the formation of MDA and 8-*iso*-PGF2α and improved the damaged activities of SOD and CAT. This indicates that early relief of carotid stenosis leads to neuronal death via inducing oxidative stress, and ischemic postconditioning could inhibit oxidative stress via improving the activities of SOD and CAT and decreasing the oxidized products of lipid and nuclear acid.

Additionally, inflammatory response is another crucial factor influencing neuronal destiny. In the brain, cytokines are expressed not only in the cells of the immune system, but are also produced by resident brain cells including neurons and glia [38]. It was reported that neurotoxic cytokines that participate in the inflammation after cerebral ischemia included interleukin-1β (IL-1β) and tumor necrosis factor-alpha (TNF-α) [39]. Thus, in this study, we examined the protein levels of IL-1β and TNF-α in the first three days under the condition of being treated with or without ischemic postconditioning when carotid stenosis was relieved. We found that relief of carotid stenosis could increase the level of IL-1β and TNF-α from day 1 to day 3, which is alleviated by ischemic postconditioning performed prior to stenosis relief. This result suggests that the protection of ischemic postconditioning is related to decreasing the higher level of IL-1β and TNF-α caused by relief of stenosis.

## 3. Experimental Section

### 3.1. Animals

Adult male Wistar rats (weighing 280–300 g) supplied by Jilin University Experimental Animal Center were housed in plastic cages (2 rats per cage) with soft bedding and free access to food and water at controlled room temperature (22–25 °C) under a 12:12 h day/night cycle, and they were kept for 7 days before the experiments. All animal procedures were approved by the ethical committee for animal experiments, Jilin University, Changchun, China. Efforts were made to minimize animal suffering and to keep the number of animals used at a minimum.

### 3.2. Surgical Procedure and Postconditioning Protocol

A method described previously [25] was used in this experiment to make severe carotid stenosis with a slight modification. Briefly, the rats were fasted overnight and freely accessed to water. Anesthesia was induced with 5% halothane and maintained with 2% halothane in oxygen/nitrous oxide (30%/70%) gas mixture. Through a midline cervical incision, the skin and muscles were bluntly dissected, and the bilateral common carotid artery was exposed and freed from its sheath. The bilateral common carotid artery was bound with a stainless microtube with a diameter of 0.45 mm and a length of 0.5 cm. Subsequently, the bilateral common carotid artery was ligated using a 4-0 suture line soaked in dexamethasone at the proximal portion that was 0.5 cm from the bifurcation of the internal and external carotid artery. During the surgical procedure, a rectal temperature was maintained between 36.5 °C and 37.5 °C. Rats were given aspirin as an anticoagulant (30 mg/L) in their drinking water 3 days after surgery.

**Figure 6:**
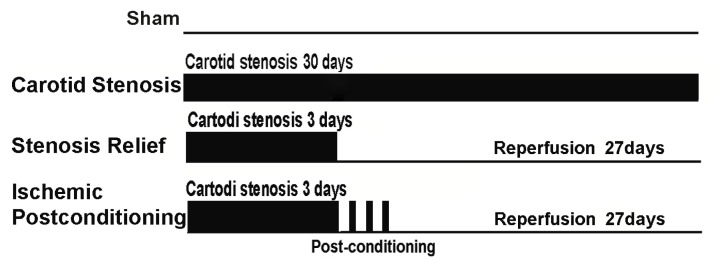
Schematic depicting the order of surgical procedures for Wistar rats undergoing sham operation, carotid stenosis, stenosis relief and ischemic postconditioning (ischemic postconditioning consisted of 3 cycles of 30-s/30-s reperfusion/clamping after 15 min ischemic episode).

At the start of the study, the rats were assigned randomly into sham-operated group, carotid stenosis group, and ischemic postconditioning group according to a computer generated randomization schedule. In the sham-operated group, the bilateral common carotid artery was exposed, but no ligature was made. In the carotid stenosis group, the carotid stenosis was maintained 3 days before it was relieved. In the ischemic postconditioning group, three cycles of 30-s/30-s reperfusion/clamping were exerted at the end of reliving carotid stenosis (Figure 6). The rats were decapitated at day 7 after the brain blood supply was reestablished.

### 3.3. Cerebral Blood Flow (CBF) Measurement

The CBF was measured with a laser Doppler flowmeter (PeriFlux System 5000, Perimed, Sweden). The rats’ scalps were opened with a scalpel when they were anesthetized with halothane. The probe holder was fixed perpendicularly to the skull at 1 mm posterior and 2.5 mm lateral to the bregma using dental resin. For CBF measurement, the probe was inserted to the probe holder. The CBF values from the rats in the sham-operated were obtained prior to surgery and 1, 3 and 5 days after the surgery. CBF was measured 2 min before both common arteries were ligated as the baseline value. CBF values were expressed as percentages relative to baseline (100%).

### 3.4. Brain Tissue Fixation

After the rat was anesthetized, the thorax was opened and the heart was disclosed. Heparin (0.1 mL, 300 IU/kg) was injected into the left ventricle before the catheter was inserted into the main artery via left atrium. Then, PBS was perfused into the vascular system at 4 °C for 3 min, and PBS with 4% paraformaldehyde was perfused at 4 °C for another 3 min. Subsequently, the brain tissue was taken out and put into PBS fixation solution containing 4% paraformaldehyde at 4 °C. Twelve hours later, the 13-μm and 50-μm coronal brain slices were cut by vibrotome and the brain slices in similarity were selected for hematoxylin-eosin (HE) staining and immunohistochemistry labeling, respectively.

### 3.5. Hematoxylin and Eosin Staining and Histological Examination

The 13-μm brain slices were mounted on slides and kept in dark room until dried. These dried slices were immersed in distilled water for 1 min prior to dehydration in gradient ethanol solution. Then, they were put into hematoxylin solution for 15 s, washed up by distilled water, immersed in Scott solution for 10 s, and washed again by distilled water. After being immersed in Eosin solution 10 s, these brain slices were redehydrated in gradient ethanol solution again, treated with dimethylbenzene and covered with coverslips. The CA1 region of each brain slice was evaluated under a light microscope and the number of intact pyramidal cells from four rats in each group was quantified at 400× magnification according to the method described previously [26].

### 3.6. Measurement of the Activities of Anti-Oxidative Enzymes

Rats were euthanized by an overdose of intraperitoneal pentobarbital, and then perfused transcardially with 100 mL icy PBS (0.1 M, pH 7.4). The bilateral hippocampus CA1 regions were isolated and homogenized in icy PBS (0.1 M, pH 7.4), and then centrifuged at 12,000 g at 4 °C for 15 min. The supernatants were collected, aliquoted, and stored at −80 °C until assay of anti-oxidative enzymes activities. The tissue protein concentration was determined by using a standard commercial kit (Bio-Rad Laboratories, Hercules, CA, USA).The activities of SOD and CAT were measured using commercial kits purchased from Cayman Chemical Company (Ann Arbor, MI, USA). Four rats were used in each experimental group for statistical analysis.

### 3.7. Measurement of Oxidative Product Level

The homogenates obtained above were also used for detecting the level of oxidative products (MDA and 8-*iso*-PGF2α). The level of MDA and 8-*iso*-PGF2α were detected respectively by specific enzyme-linked immunosorbent assay (ELISA) kits (Cayman Chemical Company, Ann Arbor, MI, USA) using a microplate reader (CA 94089, Molecular Devices, Sunnyvale, Canada). All standards and samples were run four triplicate.

### 3.8. Measurement of Inflammatory Factor Level

The tissues from hippocampus CA1 regions were homogenized in ice cold buffer (0.15 M Nacl, 5 mM EDTA, 10 mM Tris-Cl, 1% Triton X-100 and protease inhibitors cocktail). The homogenates were centrifuged (12,000× *g* for 15 min at 4 °C) and the protein concentration of the supernatants was determined as above mentioned. The concentrations of interleukin-1β (IL-1β) and tumor necrosis factor-α (TNF-α) were measured by specific enzyme-linked immunosorbent assay (ELISA) kits according to the manufacturer’s recommendations (Thermo Scientific Company, Rockford, IL, USA). All standards and samples were run four triplicate.

### 3.9. Data Analysis

The analyzers of the data were blinded to the previous procedures. All data are expressed as Mean ± SD and analyzed statistically by SPSS 17.0 software (SPSS Corp, Armonk, NY, USA). Student’s *t*-test was used as appropriate for comparison between different groups. *p* < 0.05 was considered statistically significant.

## 4. Conclusions

Our study showed that bilateral carotid stenosis could result in neuronal death in hippocampus CA1 region at 30 days. Early relief of stenosis decreases the quantity of dead neurons, but the dead neurons appear at 3 days when carotid stenosis is relieved and enhanced oxidative stress and inflammatory response are the crucial factors influencing the neuron destiny. By contrast, ischemic postconditioning performed before carotid stenosis relief could significantly rescue neuronal death via inhibition of oxidative stress and alleviating inflammatory response. Ischemic postconditioning is an effective method that could be used to protect neuronal death caused by early relief of heavy carotid stenosis.
